# Telehealth Clinical Appropriateness and Quality

**DOI:** 10.1089/tmr.2023.0019

**Published:** 2023-05-15

**Authors:** Lulu Wang, Anthony Fabiano, Arjun K. Venkatesh, Nick Patel, Judd E. Hollander

**Affiliations:** ^1^ MedStar Health, Georgetown University School of Medicine, Washington, District of Columbia, USA.; ^2^ University of Cincinnati College of Medicine, Cincinnati, Ohio, USA.; ^3^ Yale School of Medicine, New Haven, Connecticut, USA.; ^4^ University of South Carolina, Columbia, South Carolina, USA.; ^5^ Sidney Kimmel Medical College of Thomas Jefferson University, Philadelphia, Pennsylvania, USA.

**Keywords:** telehealth, clinical appropriateness, virtual examination, telehealth training, telehealth quality

## Abstract

Contrary to common perception, telehealth is not simply a substitute for in-person care. With an array of modalities—live audio–video, asynchronous patient communication, and remote patient monitoring, to name a few—telehealth creates entirely new avenues of care delivery (Table 1). Although our current care model is reactive—relying on episodic visits to an office or hospital—telehealth allows us to be proactive, filling in the gaps to provide a continuum of care. Widespread uptake of telehealth has created fertile ground for long-overdue health system reform. In this study, we describe essential next steps: redefine telehealth clinical appropriateness, evolve payment models, provide necessary training, and reimagine the patient–physician interaction.

## Redefining Clinical Appropriateness

Discussions on clinical appropriateness of telehealth frequently assume that “virtual care” exists in a category separate from “in-person care.” This nomenclature imposes a false dichotomy. Health care is health care, whether it occurs in an emergency department (ED) or through video at home. In fact, the alternative to “virtual care” is not necessarily in-person care, but often no care at all. How, then, do we create a system that delivers always-appropriate care, by guiding patients to the right avenue of care based on their individual preferences and needs?

The answer lies in digitalization. We should embrace the process that has transformed the way we interact with the world—from how we bank, to how we travel, to how we consume multimedia. Millennials have recently surpassed baby boomers as the biggest age group in our country.^1^ Increasingly, patients expect the convenience of on-demand care, while preserving access to traditional services (e.g., laboratory testing or imaging) when needed. The traditional primary care practice is familiar with reimbursement in a conventional fee-for-service model; it focuses on established patients and builds rapport through repeated in-person visits, often scheduled far in advance.

This type of care is inherently reactive: the physician manages chronic disease in a stepwise manner, with each discrete visit ingesting months of past data before adjusting the treatment regimen. There is little visibility into patient symptomatology or adherence to treatment recommendations between visits. Visit frequency is constrained by appointment availability, resulting in one-way initiation of care (“the doctor will see you now”). A patient who becomes too sick before his next appointment finds himself in the ED, and possibly admitted to the hospital. With the exception of the electronic health record and patient portal, the patient–physician interaction is “tech-lite,” and lacking in higher level digital integration.

In contrast, one can imagine a hybrid traditional–digital care system in which a patient engages with a centralized remote primary care office, which acts as an air traffic controller, orders necessary laboratories and imaging, and directs the patient to his local testing site. Once the physician receives the test results, the patient receives a communication to discuss these results and the indicated treatment—either asynchronously (by email or text) or synchronously (by phone call or two-way audio–video). This hybrid approach can appeal in particular to younger generally healthy patients who may interact sparsely with their provider due to finding in-person appointments burdensome and unnecessary.

A light-touch asynchronous tool that allows back-and-forth messaging and timely clinical guidance (smoking cessation, diet modification, and exercise) may ultimately lead to more total care delivered, and more effectively. Amazon recently purchased OneMedical and has begun offering this type of model in many cities across the United States. To further customize the patient experience, data on patient preference and customer relationship management information and physiological data through wearables can be collected.

## Payment Models and Pay Parity

Although telehealth's financial advantage is obvious in a value-based care model, most health systems, even those migrating toward digital-enabled care delivery, still rely primarily on a fee-for-service model. As a result, payment parity is a frequent topic of discussion.

As currently defined, *payment parity* for telehealth stifles innovation. Payment parity anchors virtual care services to their in-person equivalent. Rather than leveraging telehealth to reinvent the system, we are restricting care delivery to a flawed status quo. Instead of payment parity, our goal should be *value parity*, as defined by quality over cost.

Historically, both quality and value have been difficult to calculate. Quality metrics in health care have been segmented by care delivery setting, resulting in silos. For example, the Centers for Medicare and Medicaid Services (CMS), the largest user of quality measures and value-based models in the country, identifies separate quality measures for skilled nursing facilities and long-term acute care facilities, even though patients transition fluidly between the two. A patient with congestive heart failure has the same disease process regardless of his or her location, so why should the quality metrics differ?

If telehealth were to follow the same siloed blueprint, we would add “virtual care” as another care delivery setting. The result would be a cottage industry of disjointed quality measures with little attention paid to meaningful cross-segmental longitudinal measures that assess what happens in actual practice.

These divisions have prevented the world of quality measurement from achieving its quality and outcome aims. We need to think of virtual care as a population-based tool. Our goal should be to advance health outcomes and improve well-being, and to do so for the whole spectrum of care, not just within the confines of a single episode of care.

**FIG. 1.**
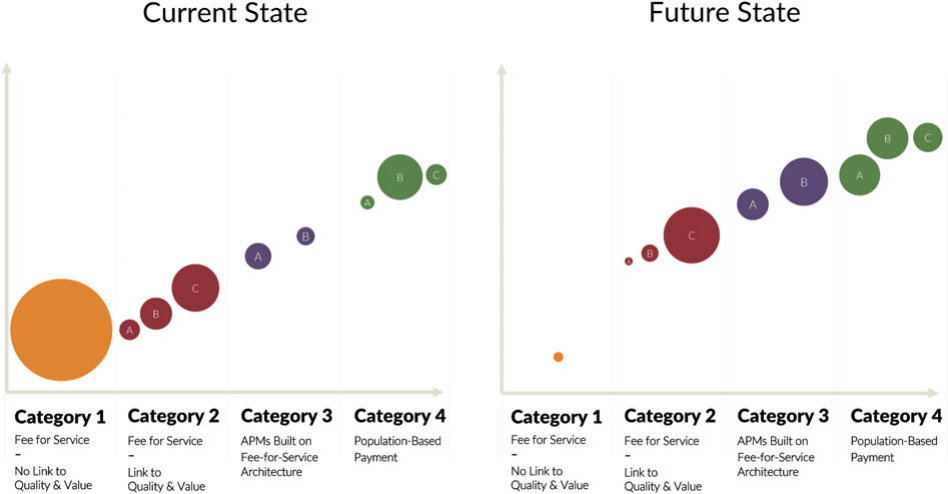
Alternative Payment Model framework (adapted from Health Care Payment Learning and Action Network White Paper, July 2017).

Figure 1, adapted from a report published by the Health Care Payment Learning and Action Network,^2^ depicts current payment models. Category 1 is an ecosystem in which all payment is fee-for-service. Most of modern health care today falls under this category. Category 2 is fee-for-service with additional year-end bonus or penalty contingent on meeting quality metrics. We have not yet defined quality and value for many specialties and procedures, making transition from Category 1 to 2 difficult. Even more, our existing quality equations rely heavily on process metrics, while lacking in outcome metrics.

Next in the progression of payment models, Category 3, comprises accountable care organizations (ACOs) and Center for Medicare & Medicaid Innovation (CMMI) models that move toward capitation and assumption of financial risk, for a defined population, by the delivery organization. Even organizations with robust virtual care programs and developed ACOs rarely exceed 40% of payments in Categories 2 and 3. Category 4 in today's landscape represents a vertically integrated health care system in which a health plan and delivery organization exist within the same entity.

Payment parity across all telehealth services pushes us toward Category 1—viewing virtual services with a purely fee-for-service lens. In doing so, the advantages of virtual care are overlooked: its ability to change the value equation by delivering high-quality care, better outcomes, and better health at a vastly different cost. Rather, we should use telehealth as a tool to advance payment reform and help organizations get from Category 2 to Categories 3 and 4.

One example of innovation in payment reform is CMMI's Emergency Triage, Transport and Treatment model.^3^ When a patient calls 911, an ambulance service provider participating in the model can facilitate a video visit at the scene (partnering with a contracted distant site provider). The goal is to identify the most appropriate location of care for the patient, whether that is treatment in place, transport to ED, or transport to a non-ED facility (e.g., skilled nursing facility or dialysis center). This interweaving of brick-and-mortar with virtual care has the potential to deliver the same quality outcome without the costs associated with transport and evaluation at a hospital. To take the model one step further, an ambulance service could partner with local health systems to assume responsibility for an entire region's population and receive capitated payment accordingly.

## Provider Training

In addition to care delivery and payment model reform, quality telehealth requires a change in provider training as well. Unlike other medical knowledge, passed from generation to generation through apprenticeship model, we cannot rely on experienced clinicians to train the less experienced or new learners. Telehealth is categorically new to most clinicians. Although the medicine remains the same, the modes of communication and the information obtained are different: clinicians can now see into the home (potentially enhancing data gathering) and clinicians cannot touch the patient (potentially impacting trust and decreasing diagnostic capabilities).

Deploying high-quality clinician training depends on two things: the desired outcome of the system and the metrics used to measure performance. If the desired outcome is to teach clinicians the mechanics of telehealth—how to conduct a video visit or request a teleconsult—these defined skills are simple to acquire. The harder concept to teach is, *how does one use telehealth as a tool to improve their patient's care experience*?

As already discussed, health care is traditionally packaged into discrete episodes. As a result, quality metrics often anchor on the individual encounter (e.g., did this virtual visit result in the right diagnosis? How much revenue was generated from this encounter? What was the patient satisfaction rating?). To know whether a virtual encounter is clinically appropriate or valuable requires us to step back and evaluate it more holistically: *how can virtual care augment traditional episodes of care and bridge the gaps between them*? The key to quality virtual care is not an acquisition of technical skill, but rather a shift in mental framing.

Once outcomes and metrics are defined, the next step is delineation of the clinician's role. First, the clinician's baseline fluency with telehealth should be assessed, including technical competency, familiarity with platform, and documentation guidelines. Despite widespread use of telehealth in the past several years, individual proficiency varies, and should be accounted for. The clinician should ensure adequate setup, including lighting, audio/video, broadband, ergonomics (e.g., external monitors), and a quiet space. Once the clinician begins practicing telehealth, he/she should receive regular feedback from more experienced telehealth providers to allow for course correction.

Transparent comparison of individual practice patterns will allow best practices to emerge. Superusers should be established within service lines to implement peer-to-peer teaching; the existing relationships between colleagues lend to personalized learning and better retention of novel telehealth practices. Clinicians should identify populations who engage well with digital tools, and lean into the diversity of telehealth services for those patients. At the same time, a clinician should know the limitations of telehealth: a referral to in-person care is not a failure of telehealth, but rather an appropriate use of telehealth as a stepping stone in the care pathway.

Like any other skill, providing virtual care improves with experience. In training, this can be done through simulation or with a standardized patient. Clinicians should practice troubleshooting audio, video, and connectivity issues and be creative with the tools at their disposal (e.g., making use of home pulse oximeters or blood pressure cuffs, or screen sharing to communicate patient education). Finally, the telehealth educator should understand that all change invokes some resistance. To provide excellent training, the educator must identify any objections and address them directly, which may require a critical reappraisal of the value or workflow of a specific telehealth program.

## Patient–Provider Interaction

Finally, we examine an evolution in the patient–provider relationship. One “standard” of patient care that has persisted throughout history is the physical examination.^4^ The embedded assumption for a telehealth patient encounter is that we should replicate the physical examination that would otherwise be done in-person. But should this be the standard for comparison? How accurate and reliable is the physical examination to begin with? If we are to develop new models of care to achieve clinical effectiveness, how *necessary* is the traditional physical examination?

Our modern understanding of the physical examination has its roots in teachings from the 18th century when Leopold Auenbrugger first described the use of percussion. An examination maneuver became the gold standard when the most senior practitioner decided it was so; there was no way to prove or disprove its correctness. Many earlier principles of the physical examination still remain core to in-person patient encounters today, but the evidence to support the accuracy or value of physical examination findings and their reliability in clinical diagnosis has been the subject of scrutiny.

Furthermore, the oft-performed templated examination is not applicable to every patient, as acknowledged by the United States Preventive Services Task Force (USPSTF), which encourages screening examinations and testing tailored to age, gender, and underlying risk.^5^

For many conditions, commonly performed maneuvers have surprisingly poor sensitivity and inter-rater reliability and have been the subject of proposed modifications or outright abandonment. A systematic review of the physical examination of patients with low-back pain found palpation of bony prominences had relatively poor inter-rater reliability for lumbar facet and sacroiliac joints, and for lumbar soft tissues.^6^ In the pulmonary examination, many findings that we routinely document such as rhonchi/rales and breath sound intensity varied widely from examiner to examiner, and pulmonologists disagreed with their *own* examinations 29% of the time.^7^ In appendicitis in children, right-sided abdominal tenderness and guarding were less reliable than a history of emesis.^8^

The concept of a physical examination with or without touch is itself a false distinction, as much of what a clinician detects through physical examination is done so before touching the patient. Examination findings that do not require touch—such as identifying a patient with increased work of breathing, change in neurological examination (e.g., abnormal speech), or the inability to abduct or externally rotate at a joint—are consistently reliable predictors of disease.^7,9,10^

Physician-guided patient self-examination demonstrates concordance with traditional physical examination and leads to the appropriate referral for abdominal imaging in patients who require urgent or emergent work-up. In a recent study, telehealth and in-person clinicians agreed on the need for imaging in 77% of 54 patients who presented to an ED for abdominal pain.^11,12^ Three patients underwent abdominal surgery within 24 h and seven patients underwent abdominal surgery within 30 days. The telehealth physician did not miss imaging for any of the patients who required surgery.

As this study of the abdominal examination suggests, the utility of the physical examination is to inform the next best intervention for the patient. If this can often be done without a “traditional” physical examination, then why is it standard practice for every patient who is seen? Advocates of the traditional physical examination maintain that it is a vital ritual and argue that failure to perform it leads to a great deal of patient dissatisfaction.^4^ However, patients have overwhelmingly expressed satisfaction with virtually performed physical examinations across a broad array of practice settings and contexts.^13^ The Mayo Clinic recently published a robust data set of >300,000 survey responses in which there was no significant difference in patient satisfaction scores between in-person visits and telehealth visits.^14^

Further contributing to the mounting evidence to support incorporation of virtual physical examination maneuvers, two recently published scoping reviews of the literature have both concluded that the virtual physical examination has similar diagnostic accuracy and is largely equivalent to the in-person examination.^13,15^ However, when considering the available data regarding the quality and value of the in-person physical examination, it raises the question of whether this should really be the gold standard in the first place. This is a topic garnering increasing attention, as leading authors in the field of digital health have begun to call for the development of a new “21st century” physical examination.^3^

As another group of authors put it, “the goal of the telehealth examination is not to directly translate the in-person exam, but to create a set of virtual exam maneuvers that provide the highest degree of actionable information to the clinician.”^15^

## Conclusion

We are in an era of actively designing the future of health care. Telehealth has the potential to revolutionize the way we care for patients (Table 1). Telehealth is not a new location of care, but a shift in mindset, and a set of tools to provide care in a way we were not before. As such, we should not relegate telehealth to the role of replicating an existing dysfunctional system. We should create condition-specific, not location-specific, quality and value metrics. We should evolve the physical examination—to re-evaluate historical maneuvers that may not add value to the care of the patient and replace them with virtual examination maneuvers can be empirically tested, taught, and adapted to fit their clinical need.

**Table 1.** Traditional Care Versus Telehealth-Enhanced Care

	Traditional care	Telehealth-enhanced care
Clinical appropriateness	Discrete, episodic care Reactive Face-to-face encounters	Continuum of care Proactive Additional modalities (remote monitoring, chat-based care)
Payment models	Fee-for-service	Contribution to value-based care
Provider training	Intergenerational Apprenticeship model (“see one, do one, teach one”)	Outcome- and competency-directed Teaching a shift in mindset (“what can hybrid care look like?”)
Physical examination	Fixed historical examination maneuvers Gold standard is in-person examination	Targeted examination Incorporation of virtual maneuvers with high sensitivity and specificity

Ultimately, we will fail if we continue holding new practices to old standards. Virtual care tools will not solve all problems all the time, but they will assist us in delivering better care when used appropriately, by appropriately trained providers, with appropriate quality metrics.

## Abbreviations Used

ACOs: accountable care organizations
APM: alternative payment model
CMMI: Center for Medicare & Medicaid Innovation
CMS: Centers for Medicare and Medicaid Services
ED: emergency department
USPSTF: United States Preventive Services Task Force
